# Consensus Definitions of Cytomegalovirus (CMV) Infection and Disease in Transplant Patients Including Resistant and Refractory CMV for Use in Clinical Trials: 2024 Update From the Transplant Associated Virus Infections Forum

**DOI:** 10.1093/cid/ciae321

**Published:** 2024-07-23

**Authors:** Per Ljungman, Roy F Chemaly, Fareed Khawaya, Sophie Alain, Robin Avery, Cyrus Badshah, Michael Boeckh, Martha Fournier, Aimee Hodowanec, Takashi Komatsu, Ajit P Limaye, Oriol Manuel, Yoichiro Natori, David Navarro, Andreas Pikis, Raymund R Razonable, Gabriel Westman, Veronica Miller, Paul D Griffiths, Camille N Kotton, for the CMV Definitions Working Group of the Transplant Associated Virus Infections Forum, Aimee Hodowanec, Takashi Komatsu, Andreas Piki, Gabriel Westman, Angie Caliendo, Sunwen Chou, Atul Humar, Parmjeet Randhawa, Monica Slavin, Michelle Wong, Dana Wolf

**Affiliations:** Department of Cellular Therapy and Allogeneic Stem Cell Transplantation, Karolinska University Hospital Huddinge, Karolinska Comprehensive Cancer Center, Stockholm, Sweden; Division of Hematology, Department of Medicine Huddinge, Karolinska Institutet, Stockholm, Sweden; Department of Infectious Diseases, Infection Control, and Employee Health, University of Texas MD Anderson Cancer Center, Houston, Texas, USA; Department of Infectious Diseases, Infection Control, and Employee Health, University of Texas MD Anderson Cancer Center, Houston, Texas, USA; Laboratoire de Bactériologie-Virologie-Hygiène, French National Reference Center for Herpesviruses, CHU Limoges, Limoges, France; Division of Infectious Diseases, Johns Hopkins, Baltimore, Maryland, USA; Merck & Co, Inc., Rahway, New Jersey, USA; Vaccine and Infectious Disease and Clinical Research Division, Fred Hutchinson Cancer Research Center, Seattle, Washington, USA; Department of Medicine, University of Washington, Seattle, Washington, USA; Takeda Pharmaceuticals Inc., Cambridge, Massachusetts, USA; Division of Antivirals, Center for Drug Evaluation and Research, US Food and Drug Administration, Silver Spring, Maryland, USA; Division of Antivirals, Center for Drug Evaluation and Research, US Food and Drug Administration, Silver Spring, Maryland, USA; Department of Medicine, Division of Allergy & Infectious Diseases, University of Washington, Seattle, Washington, USA; Infectious Diseases Service and Transplantation Center, Lausanne University Hospital and University of Lausanne, Lausanne, Switzerland; Division of Infectious Diseases, Miami Transplant Institute, Jackson Health System, University of Miami Miller School of Medicine, Miami, Florida, USA; Microbiology Service, Clinic University Hospital, INCLIVA Biomedical Research Institute, Department of Microbiology, School of Medicine, University of Valencia, Valencia, Spain; Centro de Investigación Biomédica en Red Enfermedades Infecciosas, Valencia, Spain; Division of Antivirals, Center for Drug Evaluation and Research, US Food and Drug Administration, Silver Spring, Maryland, USA; Division of Public Health, Infectious Diseases and Occupational Medicine, Department of Medicine, Mayo Clinic, Rochester, Minnesota, USA; William J. von Liebig Center for Transplantation and Clinical Regeneration, Mayo Clinic, Rochester, Minnesota, USA; Swedish Medical Products Agency, Uppsala, Sweden; Department of Medical Sciences, Section of Infectious Diseases, Uppsala University, Uppsala, Sweden; Forum for Collaborative Research, University of California, Berkeley, California, USA; Institute for Immunity and Transplantation, University College London Medical School, London, United Kingdom; Transplant and Immunocompromised Host Infectious Diseases Infectious Diseases Division, Massachusetts General Hospital, Harvard Medical School, Boston, Massachusetts, USA

**Keywords:** CMV, stem cell transplantation, organ transplantation, clinical trials, disease definitions

## Abstract

Cytomegalovirus (CMV) infection and disease are important causes of morbidity and mortality in transplant recipients. For the purpose of developing consistent reporting of CMV outcomes in clinical trials, definitions of CMV infection and disease were developed and most recently published in 2017. Since then, there have been major developments, including registration of new antiviral agents. Therefore, the Transplant Associated Virus Infections Forum, which consists of scientists, clinicians, regulators, and industry representatives, has produced an updated version of these definitions that incorporates recent knowledge with the aim of supporting clinical research and drug development. This also includes an update regarding the definition of resistant and refractory CMV infections previously published in 2019. As the field evolves, the need for updates of these definitions is clear, and collaborative efforts among clinicians, scientists, regulators, and industry representatives can provide a platform for this work.

## Teaser

The existing definitions of cytomegalovirus (CMV) infection and disease for use in clinical trials have been updated. The update is based on new developments including the introduction of clinically significant CMV infection and developments in diagnosis of CMV disease.

**(See the Editorial Commentary by Cuellar-Rodriguez and van Duin on pages 795–6.)**

Definitions of cytomegalovirus (CMV) infection and disease in patients who have undergone transplantation were initially developed and published after the 4th International CMV Conference in Paris in 1993 [1] and have been updated 3 times [2–4]. Definitions for resistant and refractory CMV infection and disease were published in 2019 [5]. These have since been used in clinical trials [6, 7] and cited more than 760 times (source: Google Scholar; accessed 10 March 2024).

Several developments have resulted in the need for another update. These definitions can also be used, when appropriate, to design trials in individuals with other severe immunocompromised conditions, such as those who have undergone Chimeric Antigen Recepter (CAR) T-cell therapy [8, 9]. Furthermore, the experience of clinical trialists who use the previous definitions, as well as from adjudication committees for such trials, has actualized the need for an update.

The Transplantation Associated Virus Infections Forum was created in 2014 (as the CMV Forum) and is based on the Forum for Collaborative Research model, which is a neutral, independent venue for all stakeholders to engage in dialogue and deliberation to advance regulatory science in disease-specific areas [10, 11]. A CMV Disease Definitions Working Subgroup reviewed the previously published documents and proposed changes based on new developments. Advice was sought from stakeholders who used these definitions in clinical trials.

## DEFINITIONS

### Definitions of CMV Infection

These definitions are unchanged from the previous version (Supplementary Material).

#### CMV Detection in Blood

It is recognized that CMV DNA detection by quantitative polymerase chain reaction (qPCR) on peripheral blood samples (“CMV DNAemia”) is associated with mortality in hematopoietic cell transplant (HCT) recipients [12]. CMV DNAemia does not necessarily mean that active CMV replication is occurring. This is supported by the detection of so-called CMV DNA blips that occur in HCT patients on letermovir prophylaxis [13] but can be found under other circumstances [14, 15]. A “blip” is characterized by single detections of CMV DNA, usually with low viral load [14, 15], and might represent either incomplete viral replication or immune system clearance of CMV.

#### Clinically Significant CMV Infection

The concept of clinically significant CMV infection has been recognized by regulatory authorities as an acceptable end point in a pivotal study of antiviral prophylaxis in HCT recipients [6]. This end point was defined as either the occurrence of CMV disease or the initiation of anti-CMV preemptive therapy based on prespecified CMV DNAemia thresholds. Currently, this end point is not acceptable in solid organ transplant (SOT) recipients.

### CMV Disease

CMV disease consists of “end-organ disease” and “CMV syndrome” in SOT recipients. To define “proven CMV end-organ disease,” the presence of appropriate clinical symptoms and/or signs is required together with documentation of CMV in tissue from the relevant organ by histopathology, virus isolation, immunohistochemistry, or nucleic acid hybridization. It is recognized that high viral DNA levels detected with quantitative nucleic acid testing in tissue or fluid obtained from the relevant organ likely represent CMV disease and might be accepted as “possible CMV end-organ disease.”

For CMV disease, it is recommended that the definitions in this article be used. For initiation of preemptive antiviral therapy, it is recommended that a standardized qPCR assay be used to measure CMV DNAemia. Unfortunately, it is difficult to define a particular viral load threshold, even when reported in international units, due to variations in patient populations, diagnostic platforms, specimen types, and viral mutations [16]. A recent publication based on a systematic review has shown that the likelihood of developing CMV disease is low, with thresholds for preemptive therapy of 2–3 log_10_ IU/mL [17]. When defining clinically significant CMV DNAemia, details of the DNAemia testing, the kinetics in viral load in serial blood samples, and the patient's risk profile for developing CMV disease should be considered.

CMV detected in blood, together with symptoms and/or signs, is not sufficient for defining end-organ disease in patients who have undergone HCT. In patients who have undergone SOT, these are included in the definitions of CMV syndrome and possible gastrointestinal (GI) disease. Methods for exclusion of other causes of the clinical syndrome need to be defined, and the symptoms need to be of a specified magnitude and clearly documented in the source documentation.

Clinical response to anti-CMV therapy might be considered as a part of a study end point. Unless CMV-specific antiviral therapy is used, however, other viral infections might also respond to a drug with broad-spectrum antiviral activity.

### Definitions of CMV Disease

Most definitions are unchanged from the previous version, and these are listed in Table 1.

**Table 1.** Definitions of Cytomegalovirus Disease Not Changed From Previous Publication

Disease Entity	Proven	Probable
Pneumonia	Clinical symptoms and/or signs of pneumonia such as new infiltrates on imaging, hypoxia, tachypnea, and/or dyspnea combined with CMV documented in lung tissue by virus isolation, rapid culture, histopathology, immunohistochemistry, or DNA hybridization techniques	
GI disease	Proven disease requires upper and/or lower GI symptoms plus macroscopic mucosal lesions plus CMV documented in tissue by histopathology, virus isolation, rapid culture, immunohistochemistry, or in situ nucleic acid hybridization techniques; studies should give information regarding the presence or absence of gut GVHD in HCT recipients	This requires upper and/or lower GI symptoms and CMV documented in tissue by histopathology, virus isolation, rapid culture, immunohistochemistry, or in situ nucleic acid hybridization but without the requirement for macroscopic mucosal lesions; it should, however, be noted that this definition may increase the risk of false positives, lowering the statistical sensitivity to a therapeutic effect; studies should report the presence or absence of gut GVHD in hematopoietic cell transplant recipients
Hepatitis	Abnormal liver function tests *plus* CMV documented in tissue by histopathology, immunohistochemistry, virus isolation, rapid culture, or DNA hybridization techniques *plus* the absence of other documented cause of hepatitis	Not defined
Encephalitis and ventriculitis	CNS symptoms *plus* detection of CMV in CNS tissue by virus isolation, rapid culture, immunohistochemical analysis, in situ hybridization, or preferably quantitative polymerase chain reaction	CNS symptoms *plus* detection of CMV in cerebrospinal fluid without visible contamination of blood *plus* abnormal imaging results or evidence of encephalitis on electroencephalogram
Nephritis	Detection of CMV by virus isolation, rapid culture, immunohistochemical analysis, or in situ hybridization in a kidney allograft biopsy specimen obtained from a patient with renal dysfunction together with the identification of histologic features of CMV infection	Not defined
Cystitis	Detection of CMV by virus isolation, rapid culture, immunohistochemical analysis, or in situ hybridization in a bladder biopsy specimen obtained from a patient with cystitis together with the identification of conventional histologic features of CMV infection	Not defined
Myocarditis	Detection of CMV by virus isolation, rapid culture, immunohistochemical analysis, or in situ hybridization in a heart biopsy specimen obtained from a patient with myocarditis together with the identification of conventional histologic features of CMV infection	Not defined
Pancreatitis	Detection of CMV by virus isolation, rapid culture, immunohistochemical analysis, or in situ hybridization in a pancreatic biopsy specimen obtained from a patient with pancreatitis together with the identification of conventional histologic features of CMV infection	Not defined
Other end-organ disease	CMV can also cause disease in other organs, and the definitions of these additional disease categories include the presence of compatible symptoms and signs and documentation of CMV by biopsy by virus isolation, rapid culture, immunohistochemical analysis, or in situ hybridization	Not defined

Abbreviations: CMV, cytomegalovirus; CNS, central nervous system; GI, gastrointestinal; GVHD, graft-versus-host disease; HCT, hematopoietic cell transplant.

#### CMV Pneumonia

*Proven Disease:* No Change

*Probable CMV pneumonia in patients after HCT:* Defined as the detection of CMV culture of bronchoalveolar lavage (BAL) fluid or the quantitation of CMV DNA in BAL fluid combined with clinical symptoms and/or signs of pneumonia in the absence of significant co-pathogens. Different threshold values have been proposed and are likely to vary among clinical settings according to patient groups, how the BAL procedure and processing are performed, and the assay used for CMV DNA quantitation. The specificity increases with a higher threshold, and the absence of CMV DNA in BAL has a very high negative predictive value against CMV pneumonia [18]. It should be recognized that CMV shedding in the respiratory tract does occur; therefore, a low CMV DNA load can represent asymptomatic infection [19].

Several studies have compared other techniques with Nucleic Acid Tests (NAT) in BAL fluid (Supplementary Table 1). The studies varied in design, the patient populations included, and how comparisons were made [18, 20–23]. There is substantial variability between different testing methods. Furthermore, no commercial assay has been validated for use on BAL fluid. Thus, it is not possible to define a specific threshold to be used in all patient categories. Despite these difficulties, for studies where qPCR is the only available method for testing BAL samples, a level of at least 3.0 log_10_ IU/mL is proposed in allogeneic HCT recipients (Table 2) based on both high positive and negative predictive values in the largest published study [18].

**Table 2.** DNA Levels in Bronchoalveolar Lavage Fluid (IU/mL) for Diagnosis of Probable or Unlikely Cytomegalovirus Pneumonia

Patient Category	Probable	Unlikely
Allogeneic HCT patients^a^	> 10^3^^a^	< 5 × 10^2^
Autologous HCT patients CAR T cell-treated patients	Cannot be defined	< 5 × 10^2^
Solid organ transplant patients except lung transplant patients isolated or combined	Cannot be defined	< 5 × 10^2^
Lung transplant patients	Cannot be defined	Cannot be defined

Abbreviation: CAR, Chimeric Antigen Receptor; HCT, hematopoietic cell transplant.

^a^Possible cytomegalovirus pneumonia is defined as the same viral load + presence of co-pathogens.

*Probable CMV pneumonia in patients after SOT:* CMV pneumonitis is a rare event in SOT recipients. Due to the lack of high-quality data for a viral load threshold from BAL samples in non-liver SOT patients, a threshold cannot be recommended at this time. However, there was consensus that a very high viral load in BAL with the “right” clinical syndrome should be captured as a probable CMV pneumonia event in clinical trials.

Biopsies are frequently done in patients who have undergone lung transplant, and the presence of CMV in tissue by immunohistochemistry with the “right” clinical syndrome is then defined as proven CMV pneumonia (Table 1). Clinical practice has developed in numerous experienced centers such that either CMV BAL fluid testing is not performed or no specific threshold is used. The variation of viral loads found in published studies is also very large (Supplementary Table 1). Thus, the recommendation is to exclude probable CMV pneumonia in lung transplant recipients as an end point in clinical trials.

*Possible CMV pneumonia:* A controversial issue in both HCT and SOT settings is how to handle the presence of co-pathogens. The detection of a concomitant primary airway pathogen together with a borderline level of CMV DNA in BAL fluid decreases the likelihood of the pneumonia being due to CMV, while a high CMV viral load in BAL fluid together with another opportunistic pathogen increases the likelihood of CMV pneumonia. It is, therefore, important to report co-pathogens in any study report. However, it is not possible to give clear guidelines on how an individual case should be handled since it depends on several factors, including the patient's immune status, other pathogen(s) detected, the CMV viral load, and the clinical picture, and is therefore defined as possible CMV pneumonia. Such cases are most appropriately handled by a case adjudication committee.

#### CMV GI Disease

*Proven disease:* No change.

*Probable disease:* No change.

*Possible GI disease in patients after SOT:* Routine clinical practice has evolved in many centers to not perform endoscopies with biopsies in nonsmall bowel SOT patients but to treat as CMV GI disease, especially in the setting of high or increasing CMV blood viral load together with clinically significant diarrhea and/or other GI symptoms in the absence of other likely causes of symptoms. In these cases, CMV DNAemia together with GI symptoms with a National Cancer Institute, Common Terminology Criteria for Adverse Events (version 4.0), intensity rating of ≥ grade 2 can be used to establish a possible diagnosis of GI CMV disease (Table 3).

**Table 3.** Definitions of Cytomegalovirus Gastrointestinal Disease

Patient Category	Proven CMV Disease	Probable CMV Disease	Possible CMV Disease
Allogeneic HCT patients	Upper and/or lower GI symptoms *plus* macroscopic mucosal lesions *plus* CMV documented in tissue^a^	Upper and/or lower GI symptoms and CMV documented in tissue^a^ (not by PCR) but without the requirement for macroscopic mucosal lesions	Upper and/or lower GI symptoms and CMV documented in tissue by PCR but without the requirement for macroscopic mucosal lesions;
Solid organ transplant patients except small bowel transplant patients	Upper and/or lower GI symptoms *plus* macroscopic mucosal lesions *plus* CMV documented in tissue^a^	Upper and/or lower GI symptoms and CMV documented in tissue (not by PCR) but without the requirement for macroscopic mucosal lesions	diarrhea rated at least National Cancer Institute, Common Terminology Criteria for Adverse Events (version 4.0), grade 2 and CMV documented in blood and exclusion of other likely causes of diarrhea
Small bowel transplant patients	Upper and/or lower GI symptoms *plus* macroscopic mucosal lesions *plus* CMV documented in tissue^a^	Not applicable	
Autologous HCT and CAR T cell–treated patients	Upper and/or lower GI symptoms *plus* macroscopic mucosal lesions *plus* CMV documented in tissue^a^	Upper and/or lower GI symptoms and CMV documented in tissue (not by PCR) but without the requirement for macroscopic mucosal lesions	

Abbreviations: CAR, Chimeric Antigen Receptor; CMV, cytomegalovirus; GI, gastrointestinal; HCT, hematopoietic cell transplant; PCR, polymerase chain reaction.

^a^Quantitative PCR is not accepted as a diagnostic criterion for proven or probable CMV GI disease.

*Rationale:* In a recent clinical trial in kidney transplant patients, there was a major discrepancy between “investigator-determined” versus “end point committee–determined” criteria for CMV GI disease reflecting on routine clinical practice [24]. Therefore, the entity of possible GI disease has been added. The presence of co-pathogens in both HCT and SOT patients is a complex issue in the absence of biopsy proof of tissue involvement. Therefore, this should be handled by a case adjudication committee.

*Possible GI disease in patients after HCT:* The detection of CMV on gut biopsies with the use of quantitative NAT was shown in 1 study to have similar sensitivity, specificity, and positive and negative predictive values as immunohistochemistry for proven and probable CMV GI disease [25]. However, due to lack of standardization and validation, the presence of upper and/or lower GI symptoms and identification of CMV in tissue by NAT without macroscopic mucosal lesions can only be used as a fulfilling criterion of possible CMV GI disease. Further development of qPCR on GI tissue (and/or noninvasive stool-based assays) should be prioritized to accurately identify GI CMV disease since it is one of the most common manifestations of end-organ CMV disease in HCT patients.

#### CMV Retinitis

Proven disease requires typical ophthalmologic exam findings for CMV retinitis plus the presence of CMV DNA in vitreous fluid or another part of the eye.

Probable disease requires typical ophthalmologic exam findings.

*Rationale:* It can be difficult to determine whether a patient has typical ophthalmological exam findings. Many centers have experience with performing sampling from vitreous fluid, allowing for consistency in the “proven” definitions that require documentation of CMV in the respective organ.

#### Other Forms of CMV End-Organ Disease

No further changes from the previous definitions are proposed (Table 1).

#### CMV Syndrome

The term “CMV syndrome” should be used only in SOT recipients and is not applicable to HCT recipients. Since it is impossible to exclude all other causes of the clinical symptomatology described as CMV syndrome, a proven category cannot be defined. It should be emphasized that careful documentation of the different symptoms is essential for confirmation of this entity, and it should be adjudicated when used in a clinical trial.

The definition of probable CMV syndrome requires detection within 1 week of CMV in blood by NAT, antigenemia, or viral culture together with at least 2 additional criteria. These are listed in Table 4.

**Table 4.** Definition of Probable Cytomegalovirus (CMV) Syndrome in Solid Organ Transplant Patients Based on Clinical and Laboratory Criteria (at Least 2 Criteria are Required) and Detection of CMV DNA or Antigen in Whole Blood or Plasma Within 1 Week of Symptoms

a. Fever ≥38°C for at least 2 days of which at least 1 measurement is documented in a healthcare setting and without another identified cause of the fever
b. New or increased malaise CTCAE toxicity grade 2, including muscle aches or general achiness, headache, or new or increased fatigue (CTCAE toxicity grade 3)
c. A WBC count of <3500/μL if the WBC count prior to the development of clinical symptoms was ≥ 4000/μL or a WBC decrease of >20% if the WBC count prior to the development of clinical symptoms was <4000/μL; the corresponding neutrophil counts are <1500/μL or a decrease of more than 20% if the neutrophil count before the onset of symptoms was below 1500/μL
d. ≥5% atypical lymphocytes
e. A platelet count of <100 000/μL if the platelet count prior to the development of clinical symptoms was ≥ 115 000/mL or a decrease of >20% if the platelet count prior to the development of clinical symptoms was <115 000/ μL
f. Elevation of hepatic transaminases (alanine aminotransferase or aspartate aminotransferase) to >2 × upper limit of normal or >2×baseline value (if abnormal at baseline); baseline defined as last value before cytomegalovirus viremia was documented (applicable to non-liver transplant recipients)

Abbreviations: CTCAE, National Cancer Institute, Common Terminology Criteria for Adverse Events (version 4.0); WBC, white blood cell.

*Rationale:* In practice, it has been difficult to verify CMV syndrome in clinical trials since adequate source documentation is frequently lacking. It is therefore emphasized that the different entities included in the syndrome definition must be verified to be accepted for the diagnosis. Because it has been especially difficult to define malaise/fatigue, the definition has been updated in the current version of the document.

### CMV Infection/Disease That Is Refractory to Treatment (With or Without Genotypic Resistance)

#### Revised Definitions of Refractory CMV Infection/Disease for Use in Clinical Trials

We consolidated the 2 categories of refractory and probable refractory CMV infection/disease into 1 category.

*Refractory CMV infection:* Defined as CMV viremia (DNAemia or antigenemia) that increases (ie, >1 log_10_ increase in CMV DNA levels in the same blood compartment from the peak viral load as measured in the same laboratory and/or with the same commercial assay) OR persists (≤1 log_10_ increase or decrease in CMV DNA levels) after at least 2 weeks of appropriate antiviral therapy.

*Refractory CMV end-organ disease:* Defined by a worsening in signs and symptoms or progression to end-organ disease (for a patient not previously diagnosed with CMV end-organ disease) OR lack of improvement in signs and symptoms after at least 2 weeks of appropriately dosed antiviral therapy.

*Rationale:* To mirror real-world practices and improve clinical trials enrollment, we consolidated the definitions of refractory and probable refractory into 1 definition for both CMV infection and end-organ disease.

*Limitations:* Certain CMV end-organ diseases (eg, CMV retinitis and GI diseases) are not always associated with measurable viral loads in serum or whole blood. In these cases, CMV may be replicating locally at tissue sites and may not be recovered for resistance testing.

#### Definition of CMV Antiviral Drug Resistance for Use in Clinical Trials

For clinical purposes, resistant CMV infection is defined as refractory CMV infection as defined above in addition to viral genetic alteration that decreases susceptibility to 1 or more antiviral drugs. Drug resistance is defined by the occurrence of viral genetic alteration that affects in vitro susceptibility and/or clinical response, typically involving genes implicated in antiviral drug anabolism (eg, UL97-mediated phosphorylation of ganciclovir [26], the antiviral drug target (eg, UL54, UL97, UL56/89/51), ATP binding (maribavir resistance mediated by UL97 mutations [27]), or compensation for antiviral inhibition of biological function (eg, UL27 [28]). There are no changes in the definitions of decreased susceptibility and viral genetic alterations that decrease drug susceptibility.

*Interpretation of mutations:* Sequence variants detected in clinical specimens have been characterized to varying degrees [29–32]. They can be categorized as follows:

- *Mutations that confer a known level of drug resistance.* Examples include the UL97 amino acid substitutions M460 V/I, H520Q, C592G, A594 V, L595S, and C603W for ganciclovir; UL54 E756 K and A809 V for foscarnet; UL54 N408 K and A987G for cidofovir; UL56 C325Y/F/W/R and V236M for letermovir; and UL97 T409M and H411Y for maribavir. In addition, certain mutations at CMV UL97, such as F342Y and C480F, confer cross-resistance to maribavir and ganciclovir that can be present before maribavir treatment, with the caveat that the C480F mutation confers a low level of resistance to ganciclovir at least in vitro [27, 33].
- *Sequence polymorphisms detected in isolates not previously exposed to antiviral drugs*, whether in vitro or in vivo. There is considerable baseline sequence polymorphism among circulating CMV strains that may affect susceptibility to antiviral drugs. While this is not fully documented for the licensed DNA polymerase inhibitors, this possibility needs ongoing evaluation, especially with newer antiviral drugs and targets. Several polymorphism studies are available for UL56, UL89, UL51, and UL27 [33, 34], and an interactive, open-access, international database for resistance and polymorphisms has been launched recently [35]; mutations that confer resistance are classified in high and low level of resistance.

## DISCUSSION

Several new interventions including antiviral drugs, vaccines, and virus-specific T cells to prevent or treat CMV infection and/or disease are in development [36–39]. For meaningful comparison of clinical outcomes, clinical studies of new agents or strategies should use common (standardized) definitions. An update of the previous definitions is warranted since transplant practices and diagnostic techniques continue to evolve. Pivotal prophylaxis studies in HCT recipients were performed with a newly defined end point (clinically significant CMV infection) [6], and this is now included in this version of the definitions.

It is unlikely that comparative studies of “old” and “new” diagnostic techniques will be performed since many diagnostic laboratories no longer use classic virus isolation or rapid culture techniques. We have addressed these developments in our definitions of probable CMV pneumonia and probable CMV retinitis. This version of CMV disease definitions has also adapted to the changes in clinical practice as seen in recent clinical trials, namely, that physicians who manage SOT patients often do not perform invasive procedures for patients with CMV detected in blood who present with diarrhea or other symptoms suggestive of GI CMV disease [24]. The classification will make it possible to find differences in outcomes between patients who develop these disease categories in future clinical trials.

In SOT recipients, the probable “CMV syndrome” category is the most frequently documented type of CMV disease. The experience from recently conducted clinical trials has been implemented and the definition somewhat changed to reflect that experience. However, CMV syndrome is not a precisely defined entity; therefore, future development could focus on a scoring system, ultimately establishing a threshold score for this entity.

This document also includes an update of the definitions of resistant and refractory CMV infection that reflects recent developments. Data support the harmful impact of refractory CMV infections with or without genotypic mutations on clinical outcomes, including mortality after SOT and HCT. The working group recognized that clinicians who care for patients at high risk for CMV-associated complications are frequently reluctant to delay changing antiviral therapy when the response is perceived to be suboptimal. Furthermore, resistance testing can take some time to obtain results in routine clinical care. However, since it takes time for many antiviral agents to achieve an antiviral effect given viral kinetics, leading to unnecessary changes in antiviral drugs that can result in negative effects on the patients, we decided that it is important to keep the previous definition of refractory infection requiring at least 2 weeks of appropriately dosed antiviral therapy.

It is important in the clinical trial setting to perform resistance testing in a structured way. The classification of mutations may evolve with addition of information, and confidence in the published data rises in proportion to the number of independent studies with the same mutation. With the introduction of new antiviral drugs for which the experience is still limited, it is important to perform resistance testing to increase the knowledge base for future patient management.

In the process of updating these definitions, it became clear that additional research is needed. Topics include a better definition of the role of CMV qPCR testing on BAL fluid in the diagnosis of CMV pneumonia, as well as the role of other factors that affect quantitation of CMV in BAL. Regarding CMV disease, use of qPCR in tissue and the role of CMV viral load in stool need to be investigated. The association of a persistently high or increasing viral load during antiviral treatment, refractory CMV infection, needs to be systematically investigated. Finally, studies are needed to understand if these definitions can be applied in other settings.

## Supplementary Data

Supplementary materials are available at *Clinical Infectious Diseases* online. Consisting of data provided by the authors to benefit the reader, the posted materials are not copyedited and are the sole responsibility of the authors, so questions or comments should be addressed to the corresponding author.

## Supplementary Material

ciae321_Supplementary_Data

## Notes

***Acknowledgments*.** The CMV Working Group consists of article authors Aimee Hodowanec, Takashi Komatsu, Andreas Piki, and Gabriel Westman and the following: Angie Caliendo, Sunwen Chou, Atul Humar, Parmjeet Randhawa, Monica Slavin, Michelle Wong, and Dana Wolf.

***Financial support*.** This work was supported by the TAVI Forum, which received funding from AiCuris, AlloVir, Evrys Bio, HHV-6 Foundation, Merck, Takeda, Symbio, Qiagen, Vera Therapeutics, and Eurofins Viracor.
