# Supplementary material for: Consensus Definitions of Cytomegalovirus (CMV) Infection and Disease in Transplant Patients Including Resistant and Refractory CMV for Use in Clinical Trials: 2024 Update From the Transplant Associated Virus Infections Forum
Source: Clin Infect Dis. 2024 Jul 23;79(3):787–94. doi: 10.1093/cid/ciae321 (PMC11426271; doi:10.1093/cid/ciae321)
Supplement: ciae321_Supplementary_Data [file ciae321_supplementary_data.docx]

***CMV Infection***: “CMV infection” is defined as virus isolation or detection of viral proteins (antigens) or nucleic acid in any body fluid or tissue specimen. It is recommended that both the source of the specimens tested (e.g., plasma, serum, whole blood, peripheral blood leukocytes (PBL), cerebrospinal fluid (CSF), bronchoalveolar lavage (BAL) fluid, urine, or tissue) and the diagnostic method used be described clearly.

***CMV Replication*:** The term replication can be used to indicate evidence of viral multiplication and is sometimes used instead of CMV infection. This term should not be used for detection of CMV DNA in peripheral blood.

***Primary CMV Infection*:** Primary CMV infection" is defined as the first detection of CMV infection in an individual who has no evidence of CMV exposure before transplantation. It is recognized that severely immunocompromised individuals such as transplant patients might not develop CMV specific antibodies.

***Recurrent CMV Infection***: “Recurrent infection” is defined as new CMV infection in a patient with previous evidence of CMV infection, which has not had virus detected for an interval of at least 4 weeks during active surveillance. Recurrent infection may result from reactivation of latent virus (endogenous) or reinfection (exogenous). It is recognized that CMV specific antibodies can be passively transferred by blood products or immune globulin administration. For practical purposes, presence or absence of CMV specific antibodies by serology can be used as acceptable estimates of previous CMV exposure to classify patients for entry into clinical trials.

***CMV Reinfection***. “Reinfection” is defined as detection of a CMV strain that is distinct from the strain that caused the initial infection.

***CMV Reactivation*.** CMV reactivation is likely if the 2 viral strains (prior and current strain) are found to be indistinguishable either by sequencing specific regions of the viral genome or by using a variety of molecular techniques that examine genes known to be polymorphic.

***Viremia*.** “Viremia” is defined as the isolation of CMV by either standard or rapid culture techniques. These techniques are, however, rarely used today for monitoring of transplant recipients. This term should not be used for detection of CMV DNA in peripheral blood.

***Antigenemia****.* “Antigenemia” is defined as the detection of CMV pp65 antigen in PBL.

***DNAemia***. “DNAemia” is defined as the detection of CMV DNA in samples of plasma, serum, whole blood, isolated PBL or in buffy-coat specimens. There are several techniques available for the detection and quantitation of CMV DNAemia. It is strongly recommended that the nucleic acid amplification techniques have been calibrated to a standard calibrator, such as the WHO International Standard for Human CMV {Fryer, 2010. #17495}, and reported in IU/ml.

***RNAemia***. “RNAemia” is defined as the detection of CMV RNA in samples of plasma, serum, whole blood, isolated PBL or in buffy-coat specimens. These techniques are not commonly used for monitoring of transplant patients despite having the theoretical advantage of documenting transcription of the genomic sequence.

**Supplementary Table 1: CMV DNA levels in BAL for definition of CMV pneumonia**

| **Patient population** | **No.** | **Suggested discriminatory viral load** | **Ref** |
| --- | --- | --- | --- |
| Various patient types including transplant patients | 1109 BAL fluids | 10,000 copies/mL | {Leuzinger, 2021 #20440} |
| Allogeneic HCT patients | 132 patients  139 controls | 200 – 500 IU/mL | {Boeckh, 2017 #20436} |
| Allogeneic HCT patients | 144 BAL fluids from 123 patients | > 500 IU/mL | {Pinana, 2019 #20437} |
| Allogeneic HCT patients | 16 allo HCT patients | 18,900 copies/mL | ([19](#_ENREF_19)) |
| Allogeneic HCT patients | 17 patients  21 controls | 34,800 IU/mL | {Beam, 2018 #20439} |
| Lung transplant patients | 66 patients  145 bronchoscopies | 4,545 IU/mL | {Lodding, 2018 #20457} |
| Lung transplant patients | 27 patients  43 BAL fluids | > 500,000 copies/mL | {Chemaly, 2005 #20458} |
| Lung transplant patients | 25 patients  39 samples with available transbronchial biopsies | 47,648 copies/mL in patients with atypical staining  1,548,827 copies/mL in patients with typical staining | {Chemaly, 2004 #20459} |

BAL: Bronchoalveolar lavage, HCT: Hematopoietic Cell Transplant; Allo: Allogeneic
